# The roles of phytogenic feed additives, trees, shrubs, and forages on mitigating ruminant methane emission

**DOI:** 10.3389/fvets.2024.1475322

**Published:** 2024-11-21

**Authors:** Ibrahim Bature, Wu Xiaohu, Xuezhi Ding

**Affiliations:** ^1^Key Laboratory of Yak Breeding Engineering, Lanzhou Institute of Husbandry and Pharmaceutical Sciences, Chinese Academy of Agricultural Sciences, Lanzhou, China; ^2^Department of Animal Science, Federal University Dutsin-Ma, Dutsin-Ma, Nigeria; ^3^Key Laboratory of Veterinary Pharmaceutical Development, Ministry of Agricultural and Rural Affairs, Lanzhou Institute of Husbandry and Pharmaceutical Sciences, Chinese Academy of Agricultural Sciences, Lanzhou, China

**Keywords:** global warming, microbial fermentation, phytogenic feed additives, rumen microbiome, rumen manipulation, rumen microbe, bioactive material

## Abstract

Ruminant animals naturally emit methane gas owing to anaerobic microbial fermentation in the rumen, and these gases are considered major contributors to global warming. Scientists worldwide are attempting to minimize methane emissions from ruminant animals. Some of these attempts include the manipulation of rumen microbes using antibiotics, synthetic chemicals, dietary interventions, probiotics, propionate enhancers, stimulation of acetogens, manipulation of rumination time, vaccination, and genetic selection of animals that produce low methane (CH_4_). The majority of synthetic additives are harmful to both beneficial rumen microbes and the host or only temporarily affect methanogenesis. Phytogenic feed additives (PFAs) have recently emerged as the best alternatives to antibiotics and synthetic chemicals because of growing public concerns regarding drug resistance and the negative impacts of antibiotics and synthetic chemicals on humans, livestock, and the environment. These additives reduce methane production and improve the volatile fatty acid profile. In this review, we provide an overview of PFA sources and how their bioactive components affect the rumen microbiome to reduce methane emissions. Additionally, we highlight the mechanisms of action of PFAs as a whole, as well as some of their bioactive components. We also review some selected trees, herbs, shrubs, and forages and their roles in reducing methane emissions.

## Graphical abstract

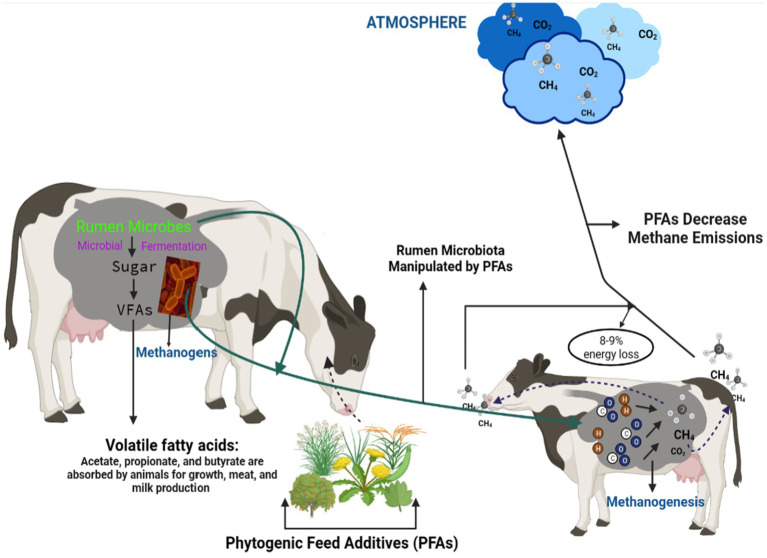

## 1 Introduction

The world’s human population is anticipated to reach almost 10 billion people by the year 2050; therefore, an increase in ruminant animal production is necessary to meet the demand for animal protein needs of humans by supplying daily meat and dairy products worldwide (1). This has resulted in the intensification of agriculture, especially livestock production, and consequently inflated the global index of methane (CH_4_) produced by livestock by almost 2.5-fold (2). Methane accounts for 16% of the global greenhouse gas emissions. It is estimated that ruminant animals contribute to 33% of the global methane emissions index (3). Approximately 81 million tons of enteric methane is produced annually by livestock worldwide. It is primarily emitted from the rumen and lower digestive tract when carbohydrates are fermented by microbes (4). These animals are among the largest producers of enteric methane, and they contribute to global warming by adding greenhouse gases to the ozone layer. This process is gaining attention worldwide for identifying rumen microbes that are important for methane production to develop the best methane mitigation strategy (5). Rumen fermentation produces a variety of beneficial products, including methane. Cattle alone contribute 15–20% of the global methane production every year (6). Methane is the most abundant hydrogen sink synthesized by methanogens in the rumen. In addition to contributing to global warming, enteric methane emissions contribute 8–9% of the total energy lost by ruminants, which, if not lost, can be used by animals for growth, meat, and milk production (7).

Most work done to reduce methane emissions in the 1950s focused on reducing feed energy loss, whereas recent efforts have focused on both energy savings and their effects on climate change. Despite the success of manipulating rumen fermentation using antibiotics and ionophores, their use has been limited by environmental and human health concerns (8). Because phytogenic feed additives (PFAs; additives derived from plants) contain many bioactive compounds, unlike antibiotics and ionophores, the global scenario has shifted toward the use of phytogenic feed additives rather than antibiotics or ionophores. This compound is capable of manipulating the microbiota in the rumen through more potent mechanisms of action, including inhibition of the activities of protozoa, methanogenic archaea, and some fiber degraders through its antimicrobial potential and decreasing hydrogen availability (9). PFAs have been reported to manipulate ruminal fermentation and to successfully reduce methane emissions from ruminants (10).

PFAs are increasingly being used in animal nutrition because of the negative effects of antibiotics and synthetic chemicals (11). These additives have sparked interest because of their potential to improve nutrient utilization and promote health (12, 13). PFA comprises various phytochemicals that are biologically active during fermentation. Various metabolic pathways are believed to mediate their antimicrobial, metabolic, immune, and antioxidant effects (14). PFAs have been tested in various ruminant models to manipulate enteric fermentation (15). The use of plant bioactive compounds (PBC) such as tannins, saponins, and essential oils for methane mitigation has been reviewed; however, most studies have focused on PBC rather than providing insight into the sources of these compounds. However, given the current trend and importance of research on climate change and global warming, more research and review are required. In this review, we explore the impact of PFAs on reducing methane emissions, with an emphasis on their effects on rumen ecology as well as the possible underlying mechanisms and factors affecting these effects.

## 2 Insight into the role of rumen microbial ecology on methanogenesis

**Figure 1:**
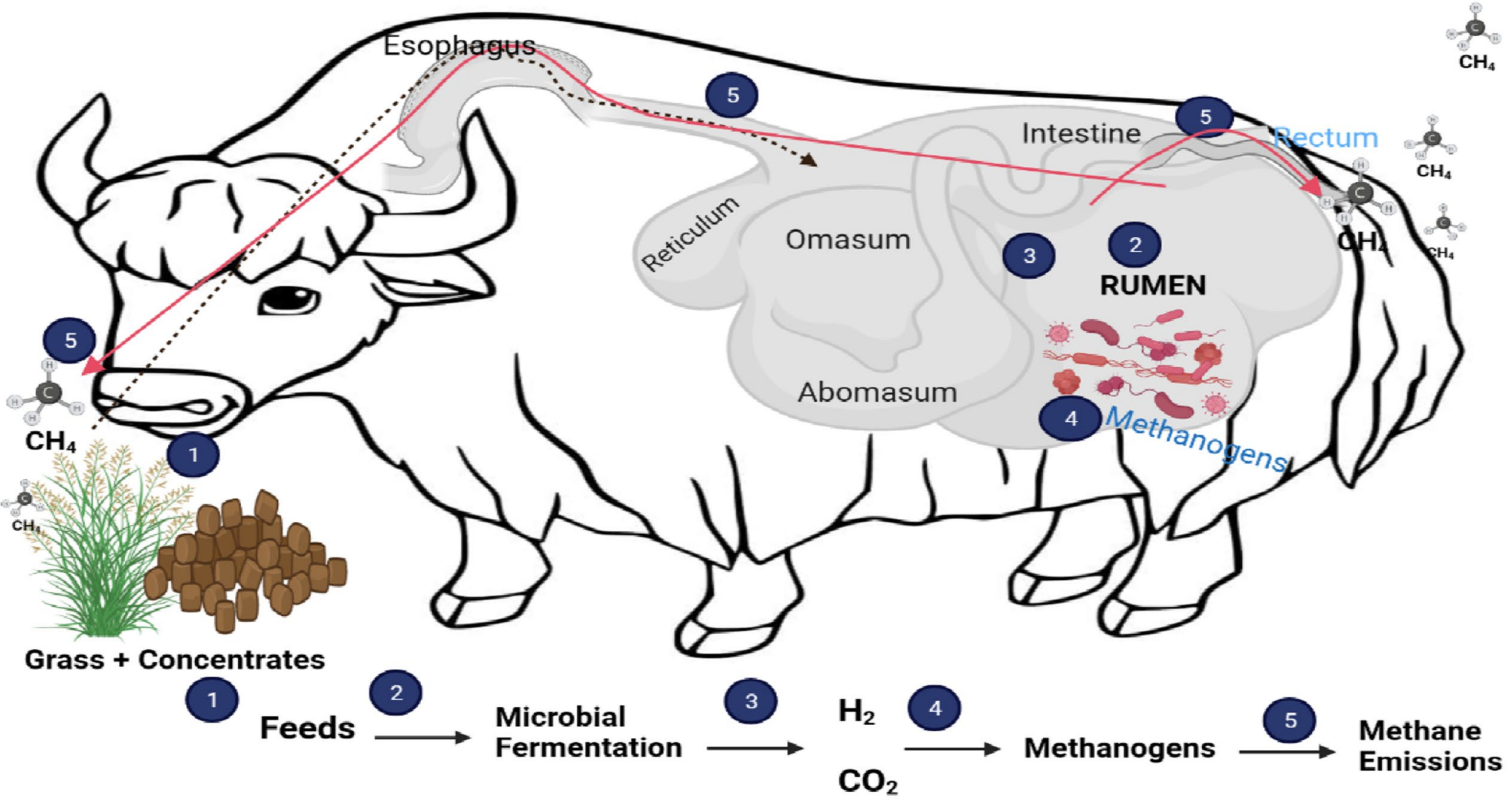
Process of methane production and role of rumen microbes.

Rumen microbes and ruminant animals have a symbiotic relationship. These microbes obtain their substrate when ruminant animals ingest feed and, in return, ferment the feed and supply valuable nutrients to the host, producing methane as a byproduct (Figure 1) (16). The microbial community in the rumen is one of the most diverse gut ecosystems hitherto described in the animal kingdom. It consists of anaerobic bacteria (10^10^–10^11^ organisms/mL), archaea (10^8^–10^9^ organisms/mL), ciliated protozoa (10^5^–10^6^ organisms/mL), anaerobic fungi (10^3^–10^4^ organisms/mL), and viral community that is largely uncharacterized (17). To date, only a few of these microbes in microbial ecologies have been cultured and characterized (18). The use of culture-based approaches to study ruminal content has decreased in recent years. However, the introduction of high-throughput sequencing techniques has allowed us to gain a better understanding of the rumen microbiome in different diets, species, and geographical locations (19). These advancements can provide a deeper understanding of the diverse microbial species in the rumen ecosystem. Using metagenomics, it will be much easier to determine which rumen microbial community is responsible for methane production. This information will enable scientists to develop the best methane mitigation strategy, which in turn will reduce the negative impacts of ruminant animals on the environment.

## 3 Phytogenic tools for reducing methane emissions and their effects on ruminal microbial ecology

Since the 18^th^ century, the loss of energy in the rumen as CH_4_ has been well-documented in a journal titled “Zeitschrift für Biologie” (Journal of Biology), written by German Scientist Tappeiner in 1884 (20). Since then, scientists worldwide have been working to reduce CH_4_ emissions without affecting livestock growth and productivity. Owing to the greenhouse gas potential of CH_4_ and the importance of ruminant contributions, policymakers worldwide are currently seeking effective mitigation strategies. In recent years, numerous studies have been conducted to reduce ruminant CH_4_ emissions (21).

**Figure 2:**
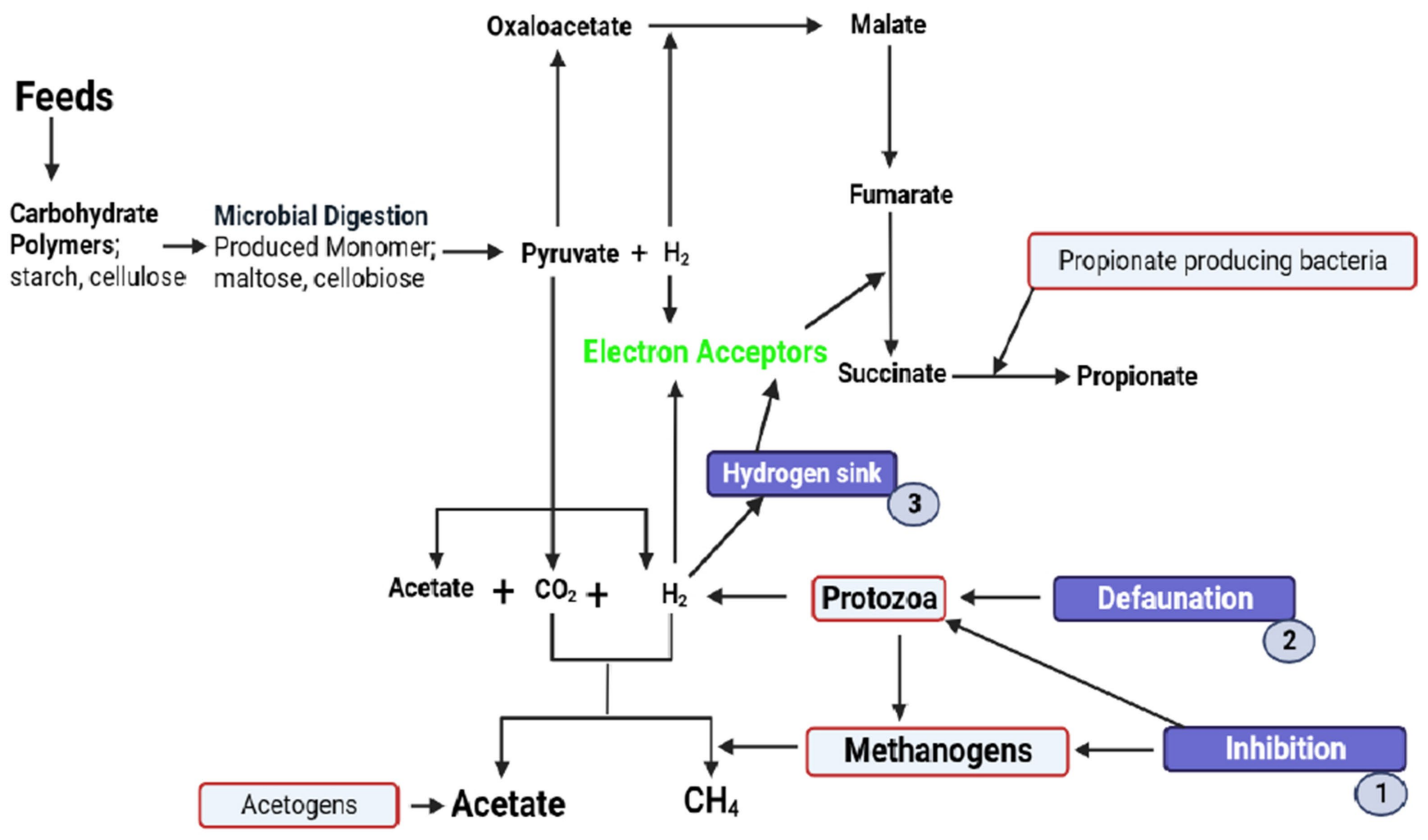
Biochemical pathways of methanogenesis affected by plant bioactive compounds to decrease methane production in the rumen. These bioactive compounds inhibit the activity of methanogens and protozoa (1). This bioactive compound decreases the number of protozoa (defaunation) (2). They also induce the rechanneling of metabolic hydrogen from CH_4_ to propionate (3).

Recently, PFAs have attracted the attention of researchers worldwide. These additives have been reported to increase feed conversion efficiency; enhance growth, productivity, and animal health; and reduce CH_4_ emissions (22). PFAs have been tested by scientists and found to significantly reduce CH_4_ emissions, manipulate rumen microbial ecology, and change the fermentation dynamics of ruminants (Figure 2) (23). These additives include; plants, part of plants, plant oil extracts, trees, shrubs, grasses, and legumes. These PFAs are rich in plant bioactive compounds (PBC) such as saponins, tannins, organosulfur compounds, essential oils, flavonoids, propolis, terpenes, and glycosides.

### 3.1 Mechanism of action of phytogenic feed additives on rumen microbial cells

**Figure 3:**
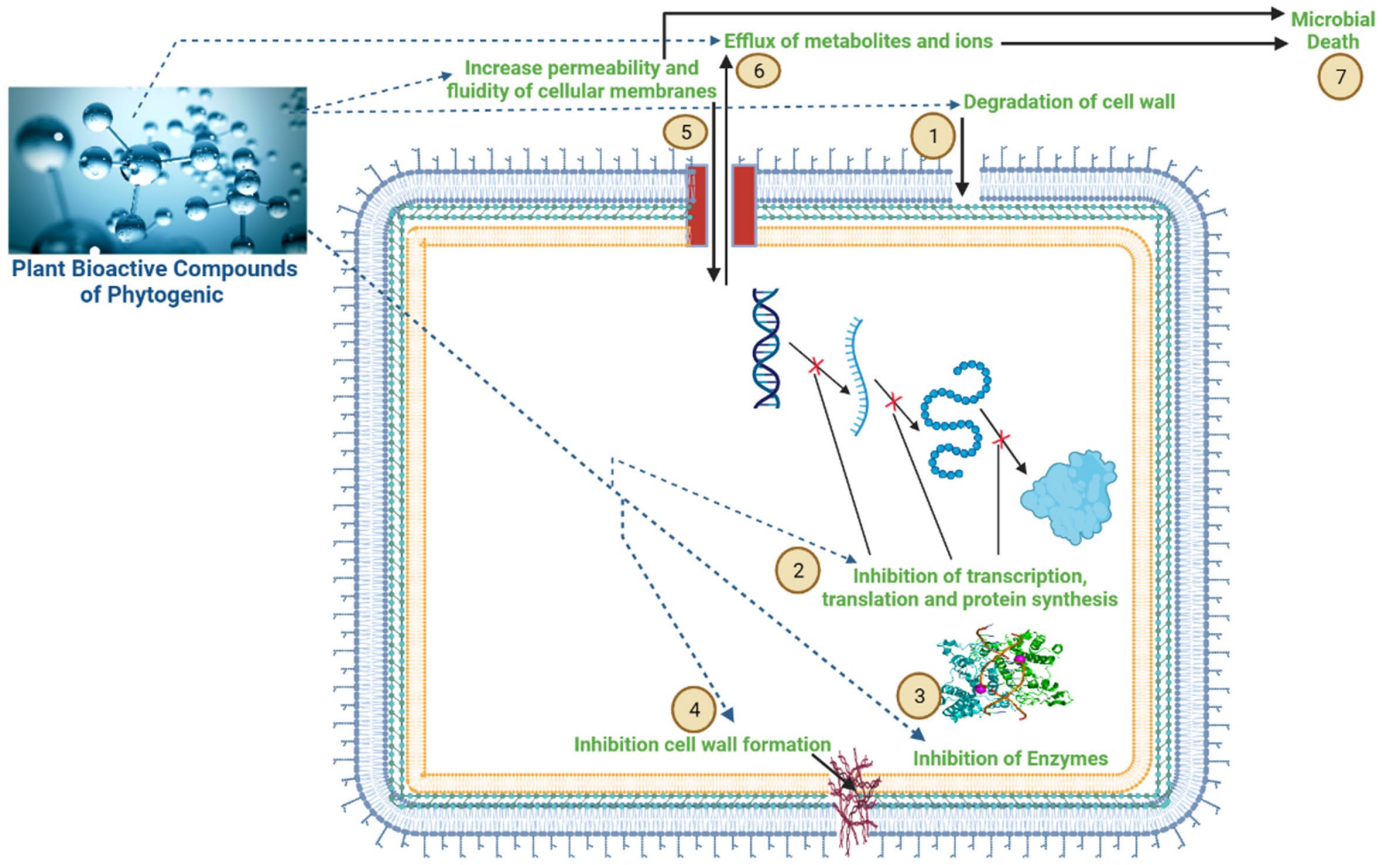
Mechanism of action of the bioactive compounds of phytogenic feed additives (PFAs) on rumen microbial cells. The bioactive compounds of phytogenic feed additives have a greater potential to modulate the ruminal microbiome and reduce CH_4_ emissions, as indicated in figure: (1) disruption of cell membranes; (2) inhibition of gene transcription, translation, and protein synthesis; (3) inhibition of enzyme activity; (4) inhibition of cell wall formation; (5) increasing permeability and fluidity of cellular membranes; (6) resulting in an influx of metabolites and ions, leading to cell leakage; and (7) microbial death.

Compared to antibiotics, PFAs have a greater potential to modify the ruminal microbiome and reduce methane emissions by disrupting cell membranes, modulating signal transduction and gene expression pathways, inhibiting enzyme activity, and inhibiting bacterial colonization (24). Generally, PFAs increase the permeability and fluidity of cellular membranes, resulting in the efflux of metabolites and ions and leading to cell leakage and microbial death (Figure 3). Moreover, they can manipulate the rumen metabolism by increasing the permeability of a specific group of rumen bacteria (25). There are several possible mechanisms of action, including disruption of the cytoplasmic membrane, disruption of the proton motive force, electron flow, active transport mechanisms, and coagulation of the cell composition (26).

### 3.2 Phytogenic feed additives: sources and effects

#### 3.2.1 Trees, shrubs, and forages

Several trees, shrubs, and forages are used for methane mitigation because they are rich in bioactive compounds that can suppress methanogenic activity. Trees, shrubs, and forages contain many bioactive compounds including terpenes, saponins, cyanogenic glycosides, flavones, isoflavones, tannins, coumarins, and other phenolics (27). In addition to these, trees, shrubs, and forages provide an excellent source of protein. Therefore, feeding trees, shrubs, and forage can be beneficial protein sources and methane reducers (28). Bioactive compounds in these plants can manipulate the rumen microbial ecosystem, thereby reducing methane production (29). The mechanism of action of these bioactive compounds could have direct effects on methanogens, anti-protozoal effects (defaunation), or inhibition of fiber digestion, followed by a lower H_2_ supply to the methanogens (Table 1).

**Table 1:** Mechanism of actions of some phytogenic feed additives sources on methane emissions.

Sources of PFAs	Plant bioactive compounds (PBC)	Mechanism of mitigation	References
*Carduus pycnocephalus*	Essential oil, Flavonoids, Terpenes	Inhibit methanogens	Bodas et al. (53)
*Paeonia lactiflora*	Total glucosides of peony	Inhibition of Gram-positive bacteria	Castillo-González et al. (58)
*Leucaena leucocephala*	Tannins	Reduction in the total number of methanogens and protozoa	Tan et al. (64)
Brassica	Glucosinolates	Alter the mean retention time of digestion in the rumen	Sun (66)
Rapeseed oil	Sterols and tocopherols	Decrease protozoal population in the rumen	Villar et al. (96).
*Camelina sativa* oil	Unsaturated fatty acids and antioxidant	Inhibiting rumen protozoa and methanogens. Hydrogen sink	Hassan et al. (15)
Garlic oil	Organosulfur	Reducing the abundance of protozoa	Kongmun et al. (108).
Palm oil	Fatty acids	Decrease the number of ciliate protozoa	Yilmaz and Kara (111)

#### 3.2.2 Trees and shrubs

##### 3.2.2.1 Gliricidia (*Gliricidia sepium*)

Gliricidia is a member of the family *Fabaceae* (legume family), subfamily *Faboideae* (*Papilionoideae*), and tribe *Robinieae*. Medium-sized semi-deciduous trees with broad canopies, native to Central America and perhaps northern South America, typically grow to a height of 10 m (occasionally 15 m) (30). *Gliricidia* foliage is rich in tannins and saponins, making it useful for mitigating methane emissions (31). The effect of long-term supplementation with *G. sepium* foliage reduced CH_4_ production in heifers, and this response persisted over time, without affecting the microbial population and VFA concentration and a slight reduction in CPD digestibility (32). Zain et al. (33) reported that 30% supplementation with *Gliricidia sepium* decreased methane gas production (from 27.22 mM to 13.13 mM) and the number of protozoa (from 6.3 × 105 cell/ml rumen fluid to 4.7 × 105 cell/mL rumen fluid) while increasing digestibility and rumen fermentation parameters. A diet supplemented with 20% *Gliricidia sepium* leaf meal has the potential to modify rumen fermentation, resulting in improved post-ruminal nutrient utilization (34).

The incorporation of *Gliricidia sepium* into animal diets reduces *in vitro* methane production and the population of ruminal protozoa (35).

##### 3.2.2.2 Calliandra (*Calliandra calothyrsus*)

Calliandra are shrubs native to the American continent that belong to the *Mimosoideae* family. These shrubs are rich in tannins (36). *Calliandra calothyrsus* is notable for its high tannin content, making it a significant candidate for mitigating methane emissions (37). Tiemann et al. (38) reported that adding tannin-rich *Calliandra* plants reduced methane emissions by 24% per day as well as per unit of feed and energy intake. They believed that the mechanism of this reduction was a reduction in the available H_2_ required for methane production by donating electrons to H_2_ to form a stable radical. *In vitro*, supplementation with *C. calothyrsus* reduces methane production without any negative effects on rumen fermentation parameters (37). According to Ridwan et al. (39), 50% silage containing *C. calothyrsus* decreased enteric CH_4_ production by reducing the total number of methanogens and goats supplemented *Methanobacteriales* but decreased bacterial diversity and organic matter digestibility. PE dairy with *C. calothyrsus* had reduced enteric methane emissions and improved milk production (40). Mwangi et al. (41) reported that replacing 40% of a protein-deficient basal diet with *Calliandra calothyrsus* reduces enteric methane emissions in both absolute terms and intensity. *Calliandra calothyrsus*, when used as a two-thirds replacement for protein in lamb diets, partially reduces methane emissions due to associated reductions in N and energy retention (38).

##### 3.2.2.3 Mulberry (*Morus alba*)

Mulberry is a fast-growing deciduous tree of the *Moraceae* family that is native to India and China’s Himalayan foothills (42). The leaves of mulberry trees are widely used as livestock feed because of their high crude protein content and metabolizable energy. In addition, they are rich in flavonoids, a plant bioactive compound known to reduce enteric methane emissions (43). *Morus alba* is significant in reducing rumen methanogenesis because it contains long-chain unsaturated fatty acids that can effectively decrease methane production in the rumen (44). The *in vitro* supplementation of mulberry leaf flavonoids at a concentration of 15 mg/100 g decreased methane emission, improved dry matter digestibility, and improved the Total Volatile Fatty Acids (TVFA) profile of sheep (45). Adding 300 g of *Morus alba* to a dairy cow’s diet changed the microbial community and fermentation process in the rumen, which increased propionate production and reduced methane emissions (46). *Morus alba* is a promising candidate for reducing enteric methane emissions while providing an optimal level of nitrogen when used as a supplement to low-quality forages (47). Considering its potential, further research is needed to test its effect on reducing methane emissions while improving the TVFA profile of ruminants.

##### 3.2.2.4 Italian plumeless thistle (*Carduus pycnocephalus*)

This plant belongs to the *Astraceae* family and genus *Carduus* (48). This plant is also popularly used in Traditional Chinese Medicine to treat various human diseases, such as colds, rheumatism, and stomachache (49). *Carduus* contains numerous classes of phytochemicals, including lignans, flavonoids, alkaloids, sterols, triterpenes, coumarins, essential oils, hexadecanoic acid, sterols, and triterpenes (50). The leaves of *C. pycnocephalus* contain tannins and saponins (51, 52). *C. pycnocephalus* decreases methane production in a hay-based diet while improving microbial protein synthesis in dairy cattle (51, 52). An *in vitro* screening of 450 plants for their potential anti-methanogenic effects concluded that *C. pycnocephalus* was the first among the six selected species, and had the potential to reduce methane emissions by more than 25% without adverse effects on digestibility, total volatile fatty acids, and gas and production (53). Owing to the antimicrobial properties of *C. pycnocephalus,* its mode of action in reducing methanogenesis may be its effect in reducing the number of rumen methanogens.

##### 3.2.2.5 Chinese peony (*Paeonia lactiflora*)

Chinese peony (*Paeonia lactiflora*) is commonly known as chishao (赤芍) in China. More than 1,200 years ago, *P. lactiflora* root was used in Traditional Chinese Medicine (54). Glucosides of Peony, or Total Glucosides of Peony (TGP), are extracted from *P. lactiflora* and contain almost 15 components, including albiflorin, benzoyl paeoniflorin, galloylpaeoniflorin, lactoferrin, oxybenzone-paeoniflorin, oxypaeoniflorin, paeony, phenol, phonolite, paeoniflorin, paeoniflorin, paeoniflorin, paeoniflorin, and paeoniflorin (55). The structures of most of these extracts are monoterpene glucosides, among which paeoniflorin is a water-soluble compound, the most abundant (>90%) has a molecular weight of 480.45 and has the highest pharmacological effects among all TGP in both *in vitro* and *in vivo* studies (54). *P. lactiflora* extracts have anti-methanogenic effect (56). *P. lactiflora* reduces methane emissions by 8–53% in cattle (57). Methane reduction is caused by the inhibition of gram-positive bacteria (58). Considering its potential antimicrobial and anti-methanogenic effects, this plant requires further investigation.

##### 3.2.2.6 Leucaena (*Leucaena leucocephala*)

The Leucaena tree belongs to the family Mimosaceae, genus; *Leucaena* and the best-known species is *Leucocephala* it has many common names Worldwide, in China, it is called “Yin ho huan” (59). Phytochemical analysis of *Leucaena* leaves revealed the presence of almost 30 compounds including tannins, squalene, phytol, phylobatanins, alkaloids, cardiac glycosides, flavonoids, saponins, and glycosides (60). Phytochemicals in Leucaena have been shown to have several anti-methanogenic effects (60). *Leucaena* decreased methane production in crossbred cows housed in an open-circuit respiration chamber (61). However, this treatment did not affect the microbial community. Supplementation with *Leucaena* decreased methane emissions by up to 20% in Colombian Lucerna heifers (62). Another 20% decrease in methane emissions has been reported in grazing cows consuming leucaena pastures in Australia (63). Leucaena is a major source of condensed tannins (CT), and *in vitro* studies of CT extracts from Leucaena resulted in 99 and 83% reductions in the total number of methanogens and protozoa, respectively (64).

#### 3.2.3 Forages

##### 3.2.3.1 Brassica forages

In temperate countries, Brassica is an annual plant that has been traditionally used in grazing systems to cover periods of feed deficits for ruminants. Brassica forage crops have four main types that are usually used worldwide to provide food for ruminant livestock feeds during shortage, this includes; kale (*Brassica oleracea* spp. *acephala*), turnips (*Brassica rapa* spp. *rapa*), swedes (*Brassica napus* spp. *napobrassica*), and forage rape (*Brassica napus* spp. *biennis*) (65). Brassica leaves, stems, bulbs, and roots are used as phytogenic additives (66). They contain bioactive compounds such as S-methyl-cysteine sulfoxide (SMCO) and glucosinolates (65). Both SMCO and glucosinolates reduce the available H_2_ for methane production through hydrogen sulfide scavenging mechanisms (67). Brassica forages were reported to reduce CH_4_ emissions in sheep by 37%; however, the experiment did not examine the effect on rumen microbial ecology (68). However, dairy cows fed Brassica forage did not show any methane mitigation effects, and the protozoal count did not significantly differ from those fed 250 g/kg DM grains as a control diet (69). This may be due to species variation or the methane measurement method used. However, Sun (66) reported that *Brassica* is rich in glucosinolates (GSLs) when ruminants consume Brassica forages, which are broken down in the rumen, resulting in absorption into the blood, which stimulates the secretion of thyroid hormone FT3 in ruminants, and the altered thyroid hormone concentration changes rumen physiology. This would alter the mean retention time of digestion in the rumen, resulting in a reduction in methane emissions.

##### 3.2.3.2 Alfalfa (*Medicago sativa* L)

Alfalfa (*Medicago sativa*), also known as lucerne, is a perennial flowering legume belonging to the *Fabaceae* family. It is the most important legume forage species in the world (70). Alfalfa is rich in saponins, which are known to reduce methane emissions. Kozłowska et al. (71) reported that ensiled Verko and Kometa alfalfa varieties (rich in saponins) reduced methane production without adversely affecting fermentation parameters. Dietary inclusion of alfalfa hay in crossbred Simmental cattle feed improves nitrogen utilization efficiency and reduces methane emissions (72). Sheep-fed alfalfa hay as a substitute for concentrate decreases CH_4_ emissions, digestibility, and urinary N and NH_4_ + -N outputs (73). A study conducted by Hironaka et al. (74) indicated that cattle fed pelleted alfalfa hay produced less methane than those fed chopped alfalfa hay.

##### 3.2.3.3 Clover (*Trifolium species*)

Clover belongs to the *Fabaceae* family, genus *Trifolium*, and has approximately 240 species distributed over the temperate and subtropical regions of the Mediterranean Basin, western North America, and eastern Africa (75). Several flavonoids, saponins, chloramines, and phenolic acids have been found in *Trifolium* plants (76). Methane yield (g/kg DM) was significantly lower in cattle-fed red clover silage (17.8 ± 3.17) than in those fed grass silage (77). A linear increase in dry matter intake (DMI) and reduced methane output per kilogram of DM consumed were observed when white clover was increased in dairy cattle diets (78). The methane yield per kilogram of dry matter and digestible organic matter intake was lower for heifers fed red and white clover silage (79). Based on *in vitro* experiments, white clover leaves containing soluble CTs of 1.6–2.4% DM reduced methane production by 19% (*p* ≤ 0.01) and ammonia production by 60% (80). Dairy cattle fed white clover pastures produce less CH_4_ than those fed ryegrass pastures in small-scale dairy systems (81). Navarro-Villa et al. (82) reported that red clover showed reduced *in vitro* rumen methane output compared to that of perennial ryegrass. In a study using portable accumulation chambers, sheep that graze subterranean clover produced lower daily CH_4_ emissions (23.5 g/day) compared with sheep grazing lucerne (27.3 g/day) and perennial ryegrass (32.3 g/day) (83).

##### 3.2.3.4 Chinese Lespedeza (*Sericea lespedeza*)

Lespedeza is a perennial herb in the family *Leguminosae* (*Fabaceae*), which is native to Japan, the Korean Peninsula, China, the Himalayas, Afghanistan, and Malaysia. Lespedeza is rich in condensed tannins and other phenolics (84). Regardless of the feeding level, goats fed CT-containing Lespedeza forage showed decreased CH_4_ emissions (85). Substituting *Eragrostis curvula* hay with 60% *S. lespedeza* on a DM basis resulted in the greatest reduction in CH_4_ yield (21.4%) compared to a diet of 100% *Eragrostis curvula* (86). Dietary inclusion of *S. lespedeza* increases propionate production and reduces CH_4_ production in the rumen (87). Liu et al. (88) reported that Alpine doelings fed on *S. lespedeza* forage emit less methane compared to the control. A study was conducted to assess nutrient digestibility, volatile fatty acid (VFA) concentrations, microbial protein synthesis, bacterial nitrogen (N) efficiency, and enteric methane (CH_4_) production in four grass-legume diets rich in condensed tannins (CT) (alfalfa, birdsfoot trefoil, crown vetch, and *S. lespedeza*). The results indicated that the lowest total CH_4_ production was observed in the *S. lespedeza* diet (89).

#### 3.2.4 Plant oil extracts

Plant oil extracts are high in lipids, making them an excellent option for mitigating methane emissions (Table 1). Numerous studies have indicated that the addition of oils to ruminant diets reduces methane production (90). This supplementation decreases the number of protozoa and methanogens in the rumen and bio-hydrogenates unsaturated fatty acids, thereby reducing methane production (91). The shift from carbohydrates to lipids in ruminant diets modifies gas production in the rumen, reducing CH_4_ emissions (92, 93).

##### 3.2.4.1 Rapeseed oil

Rapeseed is the third most popular vegetable oil in the world and is extracted from rape (canola) brassica forage. Rapeseed oil is low in erucic acid and glucosinolates (94). The inclusion of rapeseed oil in the diet of dairy cows decreased enteric CH_4_ emissions and modified the microbial community structure without affecting the total counts of bacteria, archaea, or ciliate protozoa (95). Cattle supplemented with canola oil (rapeseed oil) and nitrate reduce enteric methane emissions and protozoal populations in the rumen (96). Supplementation of nursing dairy cows with 5% rapeseed oil to nursing dairy cows reduced 23% of CH_4_ emissions with no effect on archaea and bacterial abundance (97). Dietary supplementation of rapeseed (41 g oil/kg DM) decreased daily CH_4_ emissions from lactating dairy cows by up to 22.5%, which increased the relative abundance of *Methanosphaera* and *Succinivibrionaceae* in the rumen and decreased the abundance of *Bifidobacteriaceae* (98). An *in vitro* experiment reported a decrease in the population of Thermoplasmata archaea (a methylotrophic methanogen) in the rumen after adding rapeseed oil to silage (99). Growing cattle supplemented with a diet containing 46 g of rapeseed oil/kg of diet DM decreased CH_4_ emissions, but reduced feed intake (100).

##### 3.2.4.2 *Camelina sativa* oil

*Camelina sativa* oil is a rich source of unsaturated fatty acids, particularly linoleic, linolenic, and oleic acids (101). The rich unsaturated fatty acid profile of *Camelina sativa* oil makes it a very good supplement for ruminants because it has high-quality fat (102). In addition to being rich in unsaturated fatty acids, it also has a rich antioxidant profile (102). Researchers have established that oilseeds are generally one of the most effective ways to reduce enteric CH_4_ production from ruminants, as they can mitigate CH_4_ emissions by directly inhibiting rumen protozoa and methanogens while increasing the bio-hydrogenation of polyunsaturated fatty acids to serve as a sink for hydrogen produced by rumen microbes. Supplementation with *C. sativa* oil at different levels in an *in vitro* study significantly decreased CH_4_ production (15). Camelina oil at all levels significantly affected ammonia nitrogen and microbial protein in all rations because it altered the proportions of individual ruminal volatile fatty acids and decreased CH_4_ production by altering the total number of bacteria, protozoa, and methanogens (103). Dietary supplements of camelina oil in Finnish Ayrshire cows *in vivo* decreased ruminal CH_4_ and CO_2_ production, but there was no change in the total number of bacteria, methanogens, protozoa, and fungi in the rumen (104).

##### 3.2.4.3 Garlic oil

Garlic oil was produced from ground garlic cloves and collected as a distillate from the vapor when the cloves were heated at a temperature of 100°C. Garlic oil is a mixture of various bioactive organosulfur compounds; including diallyl disulfide (C_6_H_10_S_2_), diallyl sulfide (C_6_H_10_S), allicin (C_6_H_10_S_2_O), and allyl mercaptan (C_3_H_6_S) and others (105). This metabolic profile makes it difficult to determine the exact mechanism of fermentation in the rumen. Both gram-positive and gram-negative bacteria are sensitive to the antibacterial effects of garlic oil (106). In an *in vitro* experiment, garlic oil was reported to decrease methane emissions by 74%, increase propionate and butyrate proportions, and decrease acetate and branch-chain VFA proportions (107). Garlic oils and their components have been found to modify fermentation parameters in the rumen and inhibit methane production by reducing the abundance of protozoa (108).

##### 3.2.4.4 Palm oil

Palm oil is an edible vegetable oil extracted from the fruit of palm oil trees by squeezing or crushing fleshy fruits or kernels. It contains saturated fatty acids (palmitate, 44.3%; stearate, 4.6%; myristate, 1%), monounsaturated fatty acids (oleic acid, 38.7%), and polyunsaturated fatty acids (linoleic acid, *α*-linoleic acid 10.5%, and others 0.9%) (109). Recent research on supplementation of three levels of palm oil (20, 40, and 60 g/kg) to heifers fed low-quality grass on enteric CH_4_ emissions were reported, the results show that total daily CH_4_ production decreased by 4% for every 10 g/kg palm oil added while feed conversion efficiency, apparent digestibility, intake of nutrients were not affected by the inclusion (110). It has also been reported that the addition of 4% palm oil decreases *in vitro* methane production and the number of ciliate protozoa (111).

### 3.3 Mechanism of actions of plant bioactive compounds (PBC) and their role in reducing methane emissions

PBCs are bioactive compounds that have various effects on plants and other living organisms. Many PBCs exhibit anti-methanogenic, antioxidant, antimicrobial, anti-inflammatory, anti-helminthic, anticoagulant, antidiabetic, and lipid-lowering properties (112). They are biologically active metabolites that can exert beneficial effects on methane emission, feed digestion, rumen fermentation productivity, and the health of livestock animals (113).

These compounds were extracted from the plants. These include tannins, saponins, and essential oils that affect methanogenesis by inhibiting the growth, development, and activities of the methanogen population, both directly and indirectly, by reducing the number of protozoa associated with methanogens (Table 2). They may also result in a shift toward propionate production, which reduces hydrogen competition, thereby affecting methanogenesis (23). PBC additives can be used instead of antibiotics in ruminants owing to their antibacterial properties against ruminal bacteria, protozoa, and methanogens (114). These metabolites are believed to have beneficial effects on livestock end products by altering fermentation in the rumen without causing microbial resistance (115).

**Table 2:** The effects of plant bioactive compounds (PBCs) on rumen ecology and potential mechanisms.

Plant bioactive compound	Effect on rumen ecology	Potential mechanism	References
Tannin	Bacteriostatic in rumen	Inhibit the activities of rumen microbes	McSweeney et al. (118); Jayanegara et al. (119)
	Reduce fiber digestion in the rumen.	Reduce methanogenesis by decreasing the level of available H_2_ needed for the production of methane	Patra (120); Bodas et al. (24)
	Increase in the abundance of butyrate-producing bacteria and other probiotic bacteria, such as *Bifidobacterium* and *Lactobacillusamino*	Decreased the production of short-chain fatty acids like acetate and reduced methane production	Buccioni et al. (121); Correa et al. (122)
	Suppressing the archaea communities and increasing total rumen bacteria populations	Lower methane production	Fagundes et al. (123)
	Suppressing the growth of methanogens directly	Reduce CH_4_ production	Aboagye and Beauchemin (124)
	Decreased organic matter digestion in the rumen	Reduce methanogenesis	Grainger et al. (125)
	Decreased the relative abundance of protozoa, methanogens, and *Ruminococcus albus*	Reduce methanogenesis by inhibiting methanogen and protozoal growth	Yang et al. (126); Volpe et al. (127); Witzig et al. (128)
Saponins	Inhibition of protozoal ecology in rumen and other methanogens associated with protozoa	Reduce protozoal population by interaction with sterol moiety present in the protozoa membrane thereby reducing methanogenesis	Patra and Saxena (129); Bodas and Prieto (24); Jayanegara et al. (130); Ramírez-Restrepo et al. (131); Guyader et al. (132); Liu et al. (133); Tan et al. (134)
Essential oil	Alteration of rumen microbial ecology. Inhibit the growth of methanogenic Archaea in the rumen	Inhibit the HMG-CoA reductase, which will lead to membrane instability and ultimately, death of methanogenic archaea cells. Reduce methanogenesis	Patra and Yu (135); Ye et al. (136); Lei et al. (137); Belanche et al. (138)
	Inhibition activity of gram-positive (+ve) and gram-negative (−ve) bacteria	Antimicrobial capabilities are mainly due to their interface with the cell membrane of rumen microbes by disrupting membrane stability of lips bilayers of bacterial cells. This inhibition in the rumen may lead to an increase in propionate levels in the rumen, thereby reducing the rate of methane production	Zengin and Baysal (139); Cobellis et al. (140); Schären et al. (141); Poudel et al. (142)
	Increased the abundance of *Succinivibrio species*, *Bacteroides species,* and *Succinivibrio species* in rumen.	Shift in rumen fermentation pattern, favoring propionate production over acetate. This may reduce methane production	Evans and Martin (143); Lei et al. (137)
Flavonoids	Antimicrobial properties	Their interaction with rumen microbes can decrease the population of methanogenic archaea	Purba et al. (144)
	Increase the abundance of *Fibrobacter succinogenes* diversity and decrease *Ruminoccocus albus* and *Ruminoccocus flavefaciens population*	Create a competition for hydrogen between rumen microbes and other methanogens for VFA production and methanogenesis.	Kim et al. (145)
	Reduce ciliate protozoa and hydrogenotrophic methanogens population	Inhibit methanogenesis	Oskoueian et al. (146); Seradj et al. (147)
Propolis	Reduce the population of methanogenic Archaea	Inhibit methanogenesis	Morsy et al. (148)

These phytochemicals can modify the rumen microbiome to alter its physiology because of their excellent antimicrobial activity (116). Numerous experiments on the potential effects of these phytochemicals on fermentation have been conducted both *in vitro* and *in vivo*, and have been found to significantly improve feed digestibility and decrease methanogenesis in the rumen (21). Despite various strategies to modify the microbiome of the rumen, PBC has significant potential to replace antibiotics in modifying rumen ecology and decreasing methane production through various mechanisms used by antimicrobial compounds (117). Some of the recognized mechanisms of action include disruption of proton motive force, disruption of cytoplasmic membranes, active transport mechanisms, coagulation of cell composition, and electron flow (26). PBC also significantly affects rumen microflora, resulting in the modification of fermentation and improved productivity of livestock (117).

Several PBCs, including tannins, saponins, essential oils (EO), flavonoids, and propolis have been found to have a significant impact on methanogens, protozoal population, feed conversion efficiency, absorption, and fermentation parameters as well as reducing CH_4_ emissions from animals (15) (see Table 3).

**Table 3:** Results from recent research on the effect of plant bioactive compounds (PBC) on methane emission in the rumen.

Sources of PBC	Type of experiment	Dosage	Diet	Methane emission	References
Tannins					
*Acacia mimosa* Extracts – CT	*In vivo* (6 Cannulated Nellore cattle)	1.25 and 2.25%	Grazing	28%	Fagundes et al. (123)
Extracts of Lipid encapsulated- Acacia Tannin	*In vivo* (4 rumen-cannulated Merino withers)	50 g/kg feed	Eragrotis Lucerne hay	19%	Adejoro et al. (149)
Extracts of Crude-Acacia Tannin	*In vivo* (4 rumen-cannulated Merino withers)	40 g/kg feed	Eragrotis Lucerne hay	30%	Adejoro et al. (149)
Extracts of *Acacia nilotica* Leaves and Pods	*In vitro* (Sheep rumen fluid)	Leaves (187 g/kg DM HT) Pods (350 g/kg/DM HT)	*Acacia nilotica* Leaves and Pods	64%	Rira et al. (150)
Tannin-containing – Birdsfoot trefoil, Sainfoin, and Small burnet	*In vitro* (Heifer)	2.5% CT 4.5% HT	Hay	21–34%	Stewart et al. (151)
Tannic acid	*In vivo* (Beef Cattle)	6.5, 13.0, or 26.0 g/kg DM	Corn silage and concentrate mixture	11.1, 14.7 and 33.6%	Yang et al. (126)
Purified hydrolyzable (chestnut and sumach) and Condensed tannins (mimosa and quebracho)	*In vitro (Cattle)*	0.5, 0.75 and 1.0 mg/mL	70% Hay 30% Concentrate	22–37%	Jayanegara et al. (119)
Saponins					
Tea saponin	*In vivo* (Sheep)	2.0 g/Day	Basal diet	8.8%	Liu et al. (133)
Tea saponin	*In vitro* (Bovine)	0.50 g/L	54% Corn silage 6% Hay 40% Concentrate	29%	Guyader et al. (132)
Extracts of *Yucca schidigera*	*In vivo* (Sheep)	170 mg per day	75% Hay 35% Concentrate	16%	Wang et al. (152)
Extracts of *Knautia arvensis* leaves	*In vitro* (Holstein Cow)	10.2 and 20.4 g/kg	50% Hay 50% Concentrate	5.5 and 6.4%	Goel et al. (51, 52)
Leaves of *Sesbania sesban*	*In vitro* (Holstein Cow)	174 g/kg	32% Hay: 68% Concentrate	12%	Goel et al. (51, 52)
Seeds of *Trigonella foenum-graecum*	*In vitro* (Holstein Cow)	30.4 g/kg	50% Hay 50% Concentrate	2%	Goel et al. (51, 52)
Essential oil					
Essential oil blend	*In vivo* (Dairy cow)	1 g/d/cow	Total Mixed Ration	8.8%	Belanche et al. (138)
Essential oil blend (Coriander, geranyl acetate, and eugenol)	*In vivo* (Dairy cow)	1 g/d/cow	Total Mixed Ration	6%	Hart et al. (153)
Anise oil	*In vivo* (Sheep)	0, 50, 100, 200, 400 mg/L	40% Hay 60% corn-based concentrate	47%	Wang et al. (154)
Garlic oil Eucalyptus oil Origanum oil Clove oil Peppermint oil	*In vitro* (Lactating Jersey Cow)	0.25, 0.50 and 1.0 g/L Fermentation medium	Ground alfalfa hay and concentrate 50% each	22–42% 17–26% 12–86% 11–34% 8–16%	Patra and Yu (135)

CT, Condense Tannins; HT, Hydrolysable Tannins; DM, Dry Matter Intake.

## 4 Conclusion

This review highlights various phytogenic feed additives capable of changing the rumen microbial ecology and reducing methane production. Trees, shrubs, and legumes are the most effective sources of phytogenic substances that reduce methane while improving the volatile fatty acid profile of ruminants because they contain numerous bioactive compounds. Most of the results in this review are *from in vitro* experiments; however, to understand the efficiency of phytogenic substances and their effects on methanogenesis, animal performance, animal health and welfare, rumen ecology, safety of phytogenic substances, environmental influence, quantity and quality of animal products, and applicability of phytogenic additives, *in vivo* studies over a longer period and across various parts of the world are needed. These are paramount to providing livestock farmers, policymakers, and climate change agencies with reliable information on the precise effect of phytogenic feed additives in reducing methane emissions while improving animal production.
